# Relationship between cervical lordotic angle and cervical segmental motion during craniocervical flexion test: A cross-sectional study

**DOI:** 10.1097/MD.0000000000037830

**Published:** 2024-04-12

**Authors:** Jae-hyun Lee, Ui-jae Hwang, Oh-yun Kwon

**Affiliations:** a Department of Physical Therapy, Graduate School, Yonsei University, Wonju, South Korea; b Department of Physical Therapy, College of Health Science, Laboratory of Kinetic Ergocise Based on Movement Analysis, Yonsei University, Wonju, South Korea.

**Keywords:** cervical lordosis, cervical segmental motion, craniocervical flexion test, posture, radiography

## Abstract

The craniocervical flexion test (CCFT) is commonly used for assessing the performance and function of the deep cervical flexor muscles; however, objective measurements of cervical segmental motion during craniocervical flexion (CCF) are lacking. Therefore, the purpose of this study aimed to investigate cervical segmental motions during CCFT and determine the relationship between changes of cervical segmental motions and the cervical lordotic angle. A cross-sectional study of prospectively collected data. Twenty healthy participants without neck pain underwent standing cervical radiography (lateral view) to measure the cervical lordotic angle, followed by radiography in supine position during the CCFT. The occipito-atlantal (OA) joint angle, atlantoaxial (AA) joint angle, and cervical spinous process posterior displacement (CSPPD) of the C1–C6 vertebrae were measured using lateral cervical radiographs taken during the initial (20 mm Hg) and low-stage (24 mm Hg) CCFT conditions. The CCF motion during the low-stage CCFT was characterized by a significantly increased OA joint angle, decreased AA joint angle, and increased C1–C6 CSPPD compared with the initial stage (*P* < .05). The change in the value of C1–C6 CSPPD at low-stage CCFT showed a significant positive correlation with the cervical lordotic angle. These results indicate that the cervical lordotic angle is important in minimizing CSPPD and performing appropriately-isolated CCF motion during CCFT.

## 1. Introduction

The craniocervical flexion test (CCFT) is a reliable and valid tool for evaluating the function of the deep cervical flexor muscles, such as the longus colli and longus capitis.^[1]^ In this test, a pressure biofeedback unit (PBU) is inserted between the testing surface and upper cervical spine to evaluate the flattening of the cervical lordosis associated with performing upper cervical flexion. From an average baseline pressure of 20 mm Hg, this flexion motion increases the pressure in the PBU to a maximum pressure of 30 mm Hg.^[1–3]^

The upper cervical spine consists of the occipito-atlantal (OA) and atlantoaxial (AA) joints.^[4]^ In upper cervical flexion, the convex occipital condyle rolls anteriorly and glides posteriorly within the concave superior articular surface of the C1 vertebra of the OA joint, and the inferior articular surface of C1 rolls anteriorly and glides posteriorly on the superior articular surface of C2 in the AA joint.^[2,4]^ Accordingly, the OA joint is responsible for 5° of flexion and 25° of extension motion^[2,4]^ in the upper cervical spine, whereas the AA joint allows 11° to 21° of flexion–extension motion.^[4]^ To date, there are no objective data for the cervical segmental motions occurring at the cervical spine, which would ideally require radiography usage. A study measured craniocervical flexion range of motion (ROM) during CCFT using a digital camera and custom-designed analytical software and reported that 24.9% of the full range of craniocervical flexion was used to reach the first pressure target at 22 mm Hg of the CCFT followed by linear increments up to 76.6% for the last stage of the test^[5]^; however, the authors did not describe specific proportions for segmental spinal motions occurring during CCFT.

The low-load craniocervical flexion exercise (CCFE) is used in strength training of the deep cervical flexor muscles, especially for motor control and coordination, to improve the quality of craniocervical flexion (CCF).^[6]^ The CCFE is done based on the results of the low-load exercise performed during the CCFT.^[6]^ Several studies have reported that CCFE is effective in increasing cervical upright posture,^[7]^ reducing chronic neck pain,^[6]^ and reversing the loss of cervical lordosis.^[8]^ To determine the optimum treatment approach for administering CCFE, it is important to identify movements occurring in the upper and lower cervical spine. The CCFT is focused on isolated upper cervical flexion by minimizing retraction and flexion of the middle and lower cervical spine.^[1,9]^ A previous study noted that the proportion of lower cervical spine flexion during cervical flexion was higher in participants with decreased cervical lordosis than in healthy participants.^[10]^ Moreover, the cervical lordotic angle can be affected by cervical kinematics during flexion; however, there is no evidence supporting a relationship between the cervical lordotic angle and cervical segmental motions during the CCFT. Therefore, the present study aimed to explore the motion occurring at different cervical segments during CCFT and determine the relationship between cervical lordotic angle and the change in cervical segmental motions during CCFT.

## 2. Materials and methods

### 2.1. Study participants

Based on the medical and radiological evaluation records of patients who visited the rehabilitation hospital in Wonju between January 2022 and November 2022, we recruited 20 participants without neck pain who could perform the CCFT. The participants’ cervical lordosis was pre-determined through medical records, and the cervical lordotic angle was measured using the posterior tangent technique.^[11]^ A pilot trial was conducted, which involved measuring the AA joint flexion angle at a target pressure of 24 mm Hg during CCFT. During CCFT, the electromyography amplitude of sternocleidomastoid (SCM) and anterior scalene increased from 22 to 24 mm Hg, but the magnitude of activity remained unchanged.^[12]^ Furthermore, since 24 mm Hg corresponds to 50% or less of the full range of CCF ROM and the amplitude of DCF was higher at 24 mm Hg compared to 22 mm Hg,^[5,12]^ the target pressure was set at 24 mm Hg in this study. Based on the results of the pilot trial and aiming for a power of 0.80 at an α level of 0.05, as well as partial η^2^ of 0.09, the sample size was calculated as 16 participants using the G*Power software (version 3.1.9.7; University of Trier, Trier, Germany). The sample size was increased to 20 to account for a 10% dropout rate. Participants with a history of cervical spine injury (such as instability, fracture, dislocation, facet joint pathology, or herniated disc), currently undergoing medical treatment for neck pain, or having a systemic disease related to the cervical spine (e.g., rheumatoid arthritis) were excluded from the study.^[13]^ Table 1 summarizes the characteristics of the study participants. The research protocol was approved by the Mirae Campus Institutional Review Board, Yonsei University (approval number: 1041849-202206-BM-103-02). All participants were explained the purpose of the study, and they provided informed consent before participation.

**Table 1:** Characteristics of the study participants (n = 20).

Characteristics	Mean ± SD
Age, yr	31.6 ± 4.36
Body height, cm	167.1 ± 7.81
Weight, kg	63.7 ± 10.40
Body mass index, kg/m^2^	22.73 ± 2.69
Posterior tangent, degrees	−4.85 ± 12.49

SD = standard deviation.

### 2.2. CCFT Testing procedure

**Figure 1.**
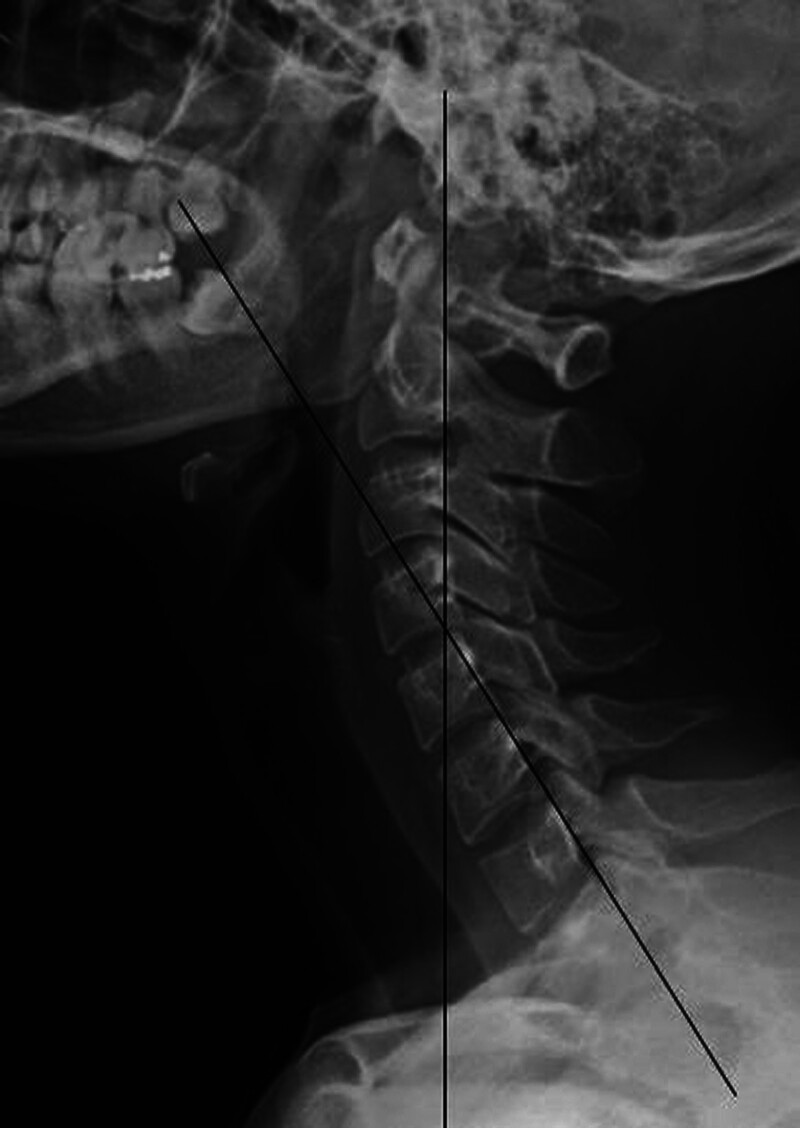
Measurement of cervical lordosis angle using the Harrison posterior tangent technique at posterior vertebral margins of C2–C7.

**Figure 2.**
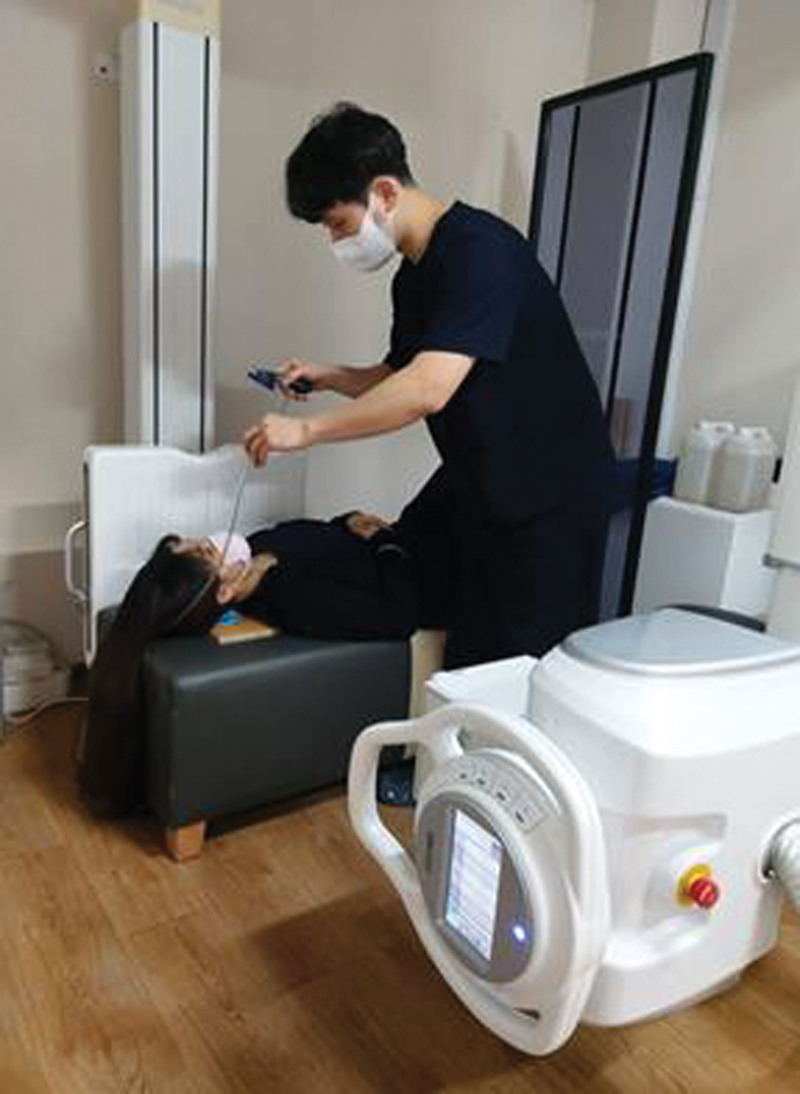
Radiographic imaging of the lateral aspect of the cervical spine during craniocervical flexion test at low stage (20–24 mm Hg).

The cervical lordotic angle was measured for all participants in standing radiographs (*R*-640-150/GXR-52S; DR Gem, Gyeonggi, Korea) using the posterior tangent technique (Fig. 1).^[11,14]^ Next, the participants were asked to perform the CCFT in a hook-lying position – hips flexed at 45° and knee flexed at 90° (Fig. 2). The principal investigator placed the PBU under the upper cervical region and inflated it to a baseline pressure of the initial test stage (20 mm Hg).^[1]^ The participants were then asked to flex the upper cervical region to increase the pressure to 24 mm Hg and maintain it for 10 seconds (low-stage CCFT). Prior practice sessions were conducted to familiarize the participants with the procedure and accustom them to the low-stage CCFT (20–24 mm Hg).^[15,16]^ Prior practice sessions ended when participants can the 10-second holds at 24 mm Hg three times without compensation.^[1]^

### 2.3. Radiological assessment

The participants stood in a relaxed position at a distance of 100 cm from the X-ray beam such that the right side of the body faced the X-ray beam, and the shadow of a metal wire was projected onto the lateral aspect of the head.^[11]^ All image analyses were performed by a radiology technologist with > 15 years of experience. The radiographic images were stored in the hospital’s picture archiving and communication system from where they were retrieved and analyzed. The cervical lordotic angle was analyzed using the posterior tangent technique of the sagittal plane,^[17]^ which represents the angle between two parallel lines drawn from the posterior wall of the vertebral bodies of C2 and C7 vertebrae; this angle provides the total cervical curvature.^[11,14]^ A posterior tangent greater than −4° indicates a decreased cervical lordotic angle, while a posterior tangent less than −4° represents a physiological cervical lordotic angle.^[11]^ Good inter- and intra-observer reliability, with a small standard error of measurement (SEM; SEM < 2.0°), has been reported for this technique.^[14]^ For acquiring the radiographs during CCFT, the participants assumed a hook-lying position in a relaxed supine position at a distance of 100 cm such that the right side of the body faced the X-ray beam, and the shadow of a metal wire was projected onto the lateral aspect of the cervical spine. To minimize parallax errors, the X-ray beam and the shadow of metal wire were kept parallel to the center of the film plate during all procedures. A wooden panel was inserted between the PBU and table surface. The uppermost line of the wooden panel was used as a reference line to measure cervical spinous process posterior displacement (CSPPD). X-rays were taken at the initial stage and low stage (24 mm Hg) of CCFT. The effective dose of the skull X-rays used in this study is 0.02 mSv (millisieverts, a unit measuring the biological effects of radiation on the human body). This value is significantly lower than the lumbar X-ray (1.5 mSv) and pelvic X-ray (0.7 mSv), indicating a low radiation dose.^[18,19]^ Moreover, it is less than the effective dose of natural background radiation (2 mSv), making the radiation exposure very minimal. X-rays taken in hospitals when patients visit for imaging typically involve an average of 4 to 6 images at various angles for each body part. Therefore, the three X-ray images used in this study have a negligible impact on the human body. The shielding efficiency of the radiation protection apron is over 95%, providing a high level of protection.^[20]^ By wearing the radiation protection apron, both participants and experimenters can reduce the radiation exposure to the body. There is no lead shielding directly applied to the skull and considering the very low effective dose on the skull, it can be inferred that the radiation protection applied to the skull has minimal impact on the human body. We provided patients with prior explanation regarding radiation exposure and obtained their consent for X-ray imaging.

### 2.4. Radiographic analysis

**Figure 3.**
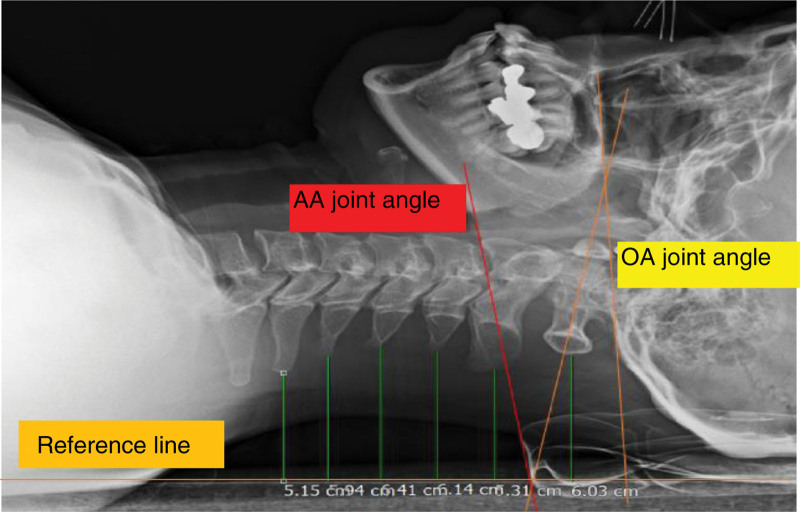
Measurement of the OA and AA joint angles and the CSPPD for C1–C6 during low stage CCFT (20–24 mm Hg) using radiography in a participant with a cervical lordotic angle of less than −4°. AA = atlantoaxial, CCFT = craniocervical flexion test, CSPPD = cervical spinous process posterior displacement, OA = occipito-atlantal.

**Figure 4.**
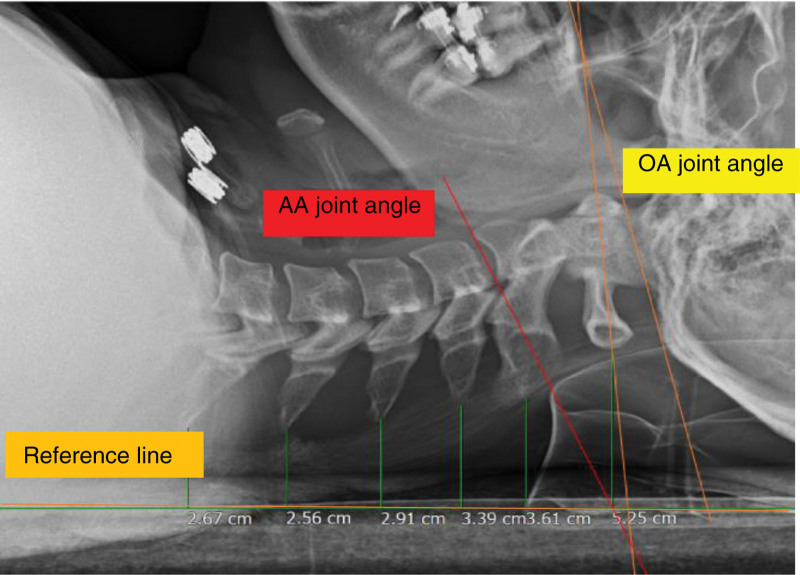
Measurement of the OA and AA joint angles and the CSPPD for C1–C6 during low stage CCFT (20–24 mm Hg) using radiography in a participant with a cervical lordotic angle of more than −4°. AA = atlantoaxial, CCFT = craniocervical flexion test, CSPPD = cervical spinous process posterior displacement, OA = occipito-atlantal.

All radiographic images were transferred to picture archiving and communication system for measuring the OA and AA joint angles and segmental CSPPD for C1–C6. The OA joint angle was defined as the sagittal plane angle between the McGregor line (posterior margin of the hard palate to the most caudal point on the outer surface of the occipital curve)^[21]^ and C1 line (formed between the middle of the anterior arch and middle of the posterior arch).^[22]^ The AA joint angle was defined as the sagittal plane angle formed between the C1 and C2 lines (lower endplate of the C2 vertebral body).^[22]^ An increase in the OA joint angle and a decrease in the AA joint angle indicated an increase in upper cervical flexion. The CSPPD for C1–C6 was measured at the initial and low stages of CCFT. CSPPD was measured as the distance between each cervical spinous process and the reference line. A decrease in CSPPD indicated an increase in cervical flexion for the corresponding cervical segment during CCFT. Figures 3 and 4 depict cervical segmental motions of the OA joint, AA joint, and C1–C6 CSPPD of individuals with different cervical lordotic angles during low-stage CCFT.

### 2.5. Statistical analysis

The Kolmogorov–Smirnov test was used to verify the normality of the data. Accordingly, a paired *t* test was used to compare the C1–C6 CSPPD, OA joint angle, and AA joint angle under both conditions (initial and low-stage CCFT). Additionally, Pearson’s correlation coefficient was used to examine the relationship between the cervical lordotic angle and OA and AA joint angles, as well as the change in the value of C1–C6 CSPPD during both CCFT conditions. All statistical analyses were performed using SPSS software (SPSS ver. 18.0 Inc., Chicago, IL) at a significance level of .05.

## 3. Results

All data were normally distributed (Kolmogorov–Smirnov test; *P* > .05). The C1–C6 CSPPD, OA joint angle, and AA joint angle at low-stage CCFT were significantly different from the values at the initial stage (*P* < .05) (Table 2). Descriptive statistics for variables and changes in cervical kinematics at low-stage CCFT are shown in Table 3.

**Table 2:** Comparison of the cervical segmental motion occurring during the initial stage (20 mm Hg) versus the low-stage (24 mm Hg) CCFT.

Variables	Initial stage	Low stage	*T*	*P*
OA joint angle	7.88 ± 4.73	10.22 ± 4.66	−6.008	<.01*
AA joint angle	22.78 ± 4.84	21.25 ± 4.87	2.484	.02*
CSPPD in C1	6.49 ± 0.99	5.86 ± 1.05	5.988	<.01*
CSPPD in C2	5.45 ± 0.95	4.88 ± 1.17	4.676	<.01*
CSPPD in C3	5.52 ± 1.23	4.96 ± 1.52	4.269	<.01*
CSPPD in C4	5.18 ± 1.37	4.68 ± 1.77	3.299	<.01*
CSPPD in C5	4.76 ± 1.48	4.34 ± 1.90	2.746	<.01*
CSPPD in C6	4.02 ± 1.71	3.68 ± 1.99	2.415	.03*

All values are presented as mean ± standard deviation.

AA = atlantoaxial, CCFT = craniocervical flexion test, CSPPD = cervical spinous process posterior displacement, OA = occipito-atlantal.

* *P* < .05.

**Table 3:** Results of the descriptive analysis for different radiographic variables and change in values of cervical segmental motions during low-stage (pressure value at 20–24 mm Hg) CCFT.

Change values	
Variables	Low stage
OA joint angle	2.3 ± 1.70
AA joint angle	−1.5 ± 2.69
CSPPD in C1	0.6 ± 0.45
CSPPD in C2	0.6 ± 0.54
CSPPD in C3	0.6 ± 0.58
CSPPD in C4	0.5 ± 0.65
CSPPD in C5	0.4 ± 0.67
CSPPD in C6	0.3 ± 0.61

All values are presented as mean ± standard deviation.

AA = atlantoaxial, CCFT = craniocervical flexion test, CSPPD = cervical spinous process posterior displacement, OA = occipito-atlantal.

Table 4 presents the results for the correlation between the cervical lordotic angle and change in the OA and AA joint angle values in the CCF and the change in values of each cervical spine flexion kinematics in C1–C6 CSPPD with pressure during low-stage CCFT. Significant positive correlations were observed between cervical lordotic angle and change in the values of CSPPD C1 (*r* = 0.527; *P* =.017), C2 (*r* = 0.581; *P* = .007), C6 (*r* = 0.589; *P* = .006), C3 (*r* = 0.609; *P* = .004), C4 (*r* = 0.628; *P* = .003), and C5 (*r* = 0.643; *P* =.002) during low-stage CCFT. However, no significant correlations were observed between the cervical lordotic angle and change in OA joint and AA joint angle values during CCF in low-stage CCFT (*P* > .05).

**Table 4:** Comparison of the Pearson correlation analysis results between change in the value of cervical segmental motions and cervical lordotic angle during low-stage (pressure value at 20–24 mm Hg) CCFT.

Variables	Cervical lordotic angle	
	*r*	*P*
Cervical lordotic angle	1	–
OA joint angle	−0.167	.483
AA joint angle	−0.322	.167
CSPPD in C1	0.527	.017*
CSPPD in C2	0.581	.007*
CSPPD in C3	0.609	.004*
CSPPD in C4	0.628	.003*
CSPPD in C5	0.643	.002*
CSPPD in C6	0.589	.006*

AA = atlantoaxial, CCFT = craniocervical flexion test, CSPPD = cervical spinous process posterior displacement, OA = occipito-atlantal.

* *P* < .05.

## 4. Discussion

To the best of our knowledge, this is the first study to investigate the relationship between cervical lordotic angle and cervical segmental motions during CCFT using radiography. Previous studies have suggested that the pressure in PBU increases gradually during CCFT corresponding to the progressive increase in performing the gentle head-nodding action of CCF.^[1,5,15]^ The deep cervical flexors, such as longus colli and longus capitis, serve as cervical stabilizers and should be targeted in the treatment of neck pain. Performing isolated CCF gradually increases PBU pressure during CCFT through the selective recruitment of the deep cervical flexors, without involving the SCM and scaleni muscles^[1,23]^; accordingly, it is important to confirm that the patient performs isolated CCF during CCFT. In this study, we observed a significant difference in cervical segmental motions while performing low-stage CCFT. In particular, the OA joint angle increased by 2.34°, the AA joint angle decreased by 1.53°, and the full ROM of CCF increased by 3.87° during low-stage CCFT compared with the initial stage.

Falla et al (2003) attached sensor markers to the tragus of the ear to measure the isolated CCF motion using digital imaging techniques and found that the full ROM of CCF during CCFT was 8.5°.^[5]^ The relative movement of the CCF at 24 mm Hg was reported to be 41.9% (which equals to 3.6°); the relative amounts of CCF motion for the 5 successive stages from 22 mm Hg to 30 mm Hg were reported to be between 24.9% and 76.6% of the full ROM.^[5]^ Notably, the difference between the CCF motion measured by Falla et al (2003) using a sensor attached to the tragus of the ear and that using radiography in the present study is only 0.3°.^[5]^ In contrast, another study that measured the ROM of the CCF at 24 mm Hg using a small wireless inertial sensor attached to the forehead reported a total of 4.65° of CCF during CCFT,^[24]^ which is a difference of 0.8° from our results. This discrepancy can be explained by the following factors. First, the use of radiography to analyze CCF motion is more accurate than the use of cutaneous sensors or digital imaging techniques as it is based on actual OA joint and AA joint angles. Second, the radiographic method employs a different analytical approach as compared with previous studies^[5,24]^ – the radiologically measured CCF motion observed in this study corroborates the increase in CCF motion observed during CCFT.^[1,6,7]^ We observed that the CCF motion was not correlated with the cervical lordotic angle; however, a significant increase in total ROM of CCF was observed in the low-stage condition compared with the initial stage, suggesting that one can perform isolated CCF regardless of the baseline cervical lordotic angle. Therefore, providing isolated CCFE to patients with impaired CCF motion, regardless of cervical lordosis, is a potentially effective treatment approach.

In a previous study, a positive correlation was observed between increasing CCF angle and increasing electromyographic amplitude of both deep cervical flexors and superficial flexor muscles.^[12]^ However, another study reported that impaired performance of the deep cervical flexor muscles prevents progressive pressure increases through the CCF, resulting in performance changes in CCFT wherein the superficial flexors were primarily used.^[25]^ The compensatory overactivation of superficial cervical flexor muscles indicates that the participant did not perform the correct movement pattern during CCFT.^[1,26]^ Individuals who have difficulty achieving progressively increasing pressure will retract their neck and flex the lower cervical spine.^[1,5]^ Jull et al (2008) reported that cervical retraction occurring during CCFT is an abnormal pattern due to poor activation of the deep cervical flexor muscles.^[1]^ However, no specific criteria for cervical retraction during CCFT has been established; thus, it is estimated as only the percentage of the full ROM of CCF using digital imaging.^[1,5,6,12]^ While our study provides valuable insights into cervical segmental motion during the CCFT, not assessing muscle activity directly through electromyography analysis is a limitation. Future research should consider incorporating electromyography to explore the relationship between muscle activity and cervical segmental motion more deeply, as this could further elucidate the mechanisms underlying effective CCFT performance and its impact on cervical lordosis.

In the present study, a significant difference in C1–C6 CSPPD was observed during the low-stage CCFT when compared with the initial stage. CSPPD decreased by 0.63 cm in C1, 0.57 cm in C2, 0.56 cm in C3, 0.5 cm in C4, 0.42 cm in C5, and 0.34 cm in C6. The decreased CSPPD during the low-stage CCFT indicates an increased cervical segmental motion. We also investigated the relationship between changing values in cervical segmental motions and cervical lordotic angle during CCFT. The decrease in the cervical lordotic angle was positively correlated with the changing values of cervical segmental motions in C1–C6 CSPPD at low-stage CCFT. The CSPPD for C3, C4, and C5 had a moderate value of ≥ 0.6, indicating that more significant changes were observed in these cervical segments compared with C1, C2, and C6. These results suggest that it is necessary to control lower cervical movements during CCFT in participants with decreased cervical lordotic angle. Figures 3 and 4 depict the differences in CSPPD between individuals with different cervical lordosis angles during low-stage CCFT. This effectively conveys the comparison between Figures 3 and 4, with Figure 4 visually illustrating a more pronounced decrease in CSPPD for a participant with a reduced cervical lordotic angle during the low-stage CCFT.

Therefore, the dual-PBU method using an additional PBU under the lower cervical region for isolated CCF motions during CCFT is recommended over the traditional CCF method.^[27]^ Training using this method reportedly increases the cross-sectional area of the longus colli muscle and decreases the muscle activity of the SCM compared with traditional CCFT.^[27]^ Therefore, when applying therapeutic intervention through CCFT in participants with a reduced cervical lordotic angle, it is necessary to control motion in the lower cervical region using methods such as dual-PBU to minimize cervical retraction when gradually increasing pressure during the low-stage CCFT. In this study, only low-stage CCFT results were analyzed; further research is warranted to investigate the movements occurring during high-stage CCFT (20–30 mm Hg).

Our study had several limitations. The study sample was small and consisted of healthy young adults aged 20 to 40 years without neck pain, which reduces the applicability of our results. Therefore, additional research is needed to investigate the relationship between cervical lordotic angle and cervical segmental motion in individuals with neck pain across different age groups during low-stage CCFT. Additionally, we measured only the angles of the OA and AA joints, without measuring the angles of the C2-C6 joints. To elucidate the relationship between cervical lordotic angle and cervical segmental motions, further investigation is necessary by examining the C2 to C6 joint angles during the CCFT. This additional research aims to prevent cervical retraction during CCFT. Furthermore, it is necessary to confirm muscle recruitment (deep cervical flexors versus superficial cervical flexors) during CCFT, which can then be correlated to cervical lordotic angle.

## 5. Conclusions

The results of this study indicate that CCFE is effective to facilitate upper cervical flexion and considering the cervical lordotic angle is important to minimize CSPPD and perform appropriate CCFT while isolating the CCF motion. Therefore, when applying CCFT or CCFE as examination and evaluation tools or therapeutic interventions for individuals with reduced cervical lordotic angle, it is necessary to control the movement in the lower cervical region to minimize cervical spine retraction.

## Author contributions

**Conceptualization:** Jae-hyun Lee, Oh-yun Kwon, Ui-jae Hwang.

**Data curation:** Jae-hyun Lee, Ui-jae Hwang.

**Formal analysis:** Jae-hyun Lee, Oh-yun Kwon, Ui-jae Hwang.

**Investigation:** Jae-hyun Lee.

**Methodology:** Jae-hyun Lee, Ui-jae Hwang.

**Project administration:** Oh-yun Kwon.

**Supervision:** Oh-yun Kwon, Ui-jae Hwang.

**Validation:** Oh-yun Kwon, Ui-jae Hwang.

**Visualization:** Jae-hyun Lee.

**Writing – original draft:** Jae-hyun Lee, Ui-jae Hwang.

**Writing – review & editing:** Jae-hyun Lee, Oh-yun Kwon, Ui-jae Hwang.
